# Potential Role of Ribonucleotide Reductase Enzyme in Mitochondria Function and Woody Breast Condition in Broiler Chickens

**DOI:** 10.3390/ani13122038

**Published:** 2023-06-20

**Authors:** Majid Shakeri, Byungwhi Kong, Hong Zhuang, Brian Bowker

**Affiliations:** U.S. National Poultry Research Center, USDA-ARS, Athens, GA 30605, USA; byungwhi.kong@usda.gov (B.K.); hong.zhuang@usda.gov (H.Z.)

**Keywords:** broiler chickens, woody breast, ribonucleotide reductase activity, mitochondria

## Abstract

**Simple Summary:**

The woody breast myopathy in broiler chickens results in inferior quality breast meat. The severity of woody breast can negatively impact consumers’ acceptance, resulting in significant economic loss to the industry each year. The underlying biological mechanisms that cause woody breast development are not well understood. This study demonstrated that ribonucleotide reductase (an enzyme necessary for DNA synthesis and repair) and genes related to mitochondria function reduced for woody breast, while woody breast increased fibrosis, suggesting a possible pathway for woody breast to impair energy production in the affected muscle. As direct mechanistic connections between ribonucleotide reductase and woody breast have not been established before, further studies are required to provide a better understanding of how ribonucleotide reductase is involved in woody breast development.

**Abstract:**

The cellular events leading to the development of the woody breast myopathy in broiler breast muscle are unclear. Affected woody breast muscle exhibits muscle fiber degeneration/regeneration, connective tissue accumulation, and adverse morphological changes in mitochondria. Ribonucleotide reductase (RNR) is an enzyme for the synthesis of dNTP, which is important for mitochondria DNA content (mtDNA). RNR consists of two subunits: *RRM1/RRM2*. A decrease in *RRM2* is associated with a decrease in mtDNA and mitochondria proteins, leading to impaired ATP production. The objective of this study was to investigate potential RNR differences between woody breast (WB) and normal (N) breast muscle by examining *RRM2* expression and associated pathways. Gene expression and enzyme activities were examined by qPCR and commercial kits. Results showed that *RRM2* expression reduced for WB (*p* = 0.01) and genes related to mitochondria, including *ATP6* (*p* = 0.03), *COX1* (*p* = 0.001), *CYTB* (*p* = 0.07), *ND2* (*p* = 0.001) and *ND4L* (*p* = 0.03). Furthermore, *NDUFB7* and *COX 14*, which are related to mitochondria and ATP synthesis, tended to be reduced in WB. Compared to N, *GLUT1* reduced for WB (*p* = 0.05), which is responsible for glucose transport in cells. Consequently, *PDK4* (*p* = 0.0001) and *PPARG* (*p* = 0.008) increased in WB, suggesting increased fatty acid oxidation. Citric synthase activity and the NAD/NADH ratio (*p* = 0.02) both reduced for WB, while WB increased *CHRND* expression (*p* = 0.001), which is a possible indicator of high reactive oxygen species levels. In conclusion, a reduction in *RRM2* impaired mitochondria function, potentially ATP synthesis in WB, by increasing fibrosis and the down-regulation of several genes related to mitochondria function.

## 1. Introduction

Woody breast is an abnormal muscle condition that negatively impacts the texture and appearance of chicken breast meat. The specific cause is unknown, but broilers have been intensely selected for high breast meat yield, which is considered to be one of the factors associated with the development of woody breast [1]. The myopathies can negatively impact consumers’ acceptance, resulting in significant economic loss to the industry reaching > $1 billion/year of loss globally [2].

Although woody breast meat is wholesome and safe for consumers, the texture of cooked woody breast meat is tough and rubbery [1,3], which is likely the result of the high content of connective tissue and damaged muscle fiber structure observed in the affected muscle. Indicators of the woody breast condition in the pectoralis muscle can be detected at early ages in broilers, which suggests the problem might be genetic [3]. Several studies in recent years have focused on genes related to woody breast meat development [4]. Studies associated the woody breast meat problem with mitochondria function [5] and energy (ATP) production [6]. The mitochondria are involved in cells’ oxygen consumption and generate reactive oxygen species (ROS). Woody breast muscles have higher ROS localized in the outer mitochondria membrane; therefore, the affected muscles exhibit mitochondria dysfunction [7], leading to oxidative stress due to low oxygen supply, which in turn causes muscle damage [8]. Mitochondria dysfunctions have been known to be responsible for several disorders and abnormal cell functions [9]. Impaired oxidative phosphorylation (vital reaction for the cellular storage and transfer of free energy) leading to a decrease in cellular ATP production is the most important factor underlying mitochondria dysfunction in associated diseases [7,9,10]. An enzyme that is involved in ATP production and mitochondria function that has not been studied in woody breast is ribonucleotide reductase (RNR).

Ribonucleotide reductase is a key enzyme that converts ribonucleotides to deoxyribonucleotides, which are the building blocks for DNA replication and repair in every living cell [11]. RNR consists of two subunits encoded by genes: ribonucleotide reductase M1 subunit catalytic (*RRM1*) and ribonucleotide reductase M2 subunit regulatory (*RRM2*). The *RRM2* acts as a molecular switch that affects RNR activity and DNA replication [12]. Decreases in *RRM2* expression are associated with severe reductions in mitochondria DNA content (mtDNA), leading to impaired ATP production in various tissues [13] and cellular function [14]. Furthermore, reduced mtDNA could have an adverse impact on the levels of mitochondria gene transcripts, resulting in a decreased amount of abundance of mitochondria proteins. The strict control of RNR activity is critical, as decreased RNR activity induces replication anomalies and genome instability. Thus, RNR activity is finely regulated allosterically and at the transcriptional level [11,15]. In fact, RNR, especially the *RRM2* subunit, is critical for cell division and ATP production. Thus, insufficient RNR activity resulted in aberrant DNA replication, decreased mitochondria function and increased cell lethality [16]. However, current gaps in knowledge include understanding if RNR has an essential role in woody breast meat and whether altering RNR activity may play a role in woody breast incidence. Therefore, the objective of this study was to investigate potential RNR differences between normal and woody breast muscle by examining *RRM2* expression and associated pathways.

## 2. Materials and Methods

### 2.1. Samples

Breast muscles (Ross-308, ~8 weeks old) were obtained (~3 h post-mortem) from the deboning line of a commercial broiler processing plant where birds were slaughtered according to standard industry procedures. The collection time was based on the commercial practice and previously published works that showed there is no significant changes in RNA purity up to ~48 h post-mortem [17,18], and RNA degradation happens at 48 h post-mortem. Whole breast fillets were scored and divided to two groups: severe woody (WB) and normal (N) breast meat. A total of 8 WB and 8 N breast muscles were selected for analysis. Scoring was performed by a panel of experts according to published criteria [19] as follows: normal = flexible throughout and severe = extremely hard throughout from the cranial region to the caudal tip. Muscles samples were removed from the cranial end of fillets, fixed in liquid nitrogen, and stored at −80 °C for subsequent analysis of enzyme activities and gene expression.

### 2.2. Enzyme Activities

The NAD/NADH ratio and citrate synthase activity (CS) were measured using commercial kits (Sigma Aldrich, St. Louis, MO, USA) based on the manufacturer’s instructions. The supernatant obtained from homogenized muscle tissues was used to measure NAD/NADH and CS levels at 450 nm and 412 nm, respectively.

Briefly, for CS assay, ~20 mg tissue rapidly homogenized with 100 mL of citrate assay buffer (provided by the manufacturer). Afterwards, it was centrifuged at 15,000× *g* for 10 min to obtain the supernatant. Then, 50 mL of the provided reaction mix was added to each of the standard (provided by the manufacturer), sample, and blank control wells. They were mixed well using a horizontal shaker and incubated at room temperature for 30 min in a dark place before measuring at 412 nm. For NAD/NADH assay, ~20 mg of tissue was washed with cold PBS. The samples were homogenized with extraction buffers (provided by the manufacturer) for NAD and NADH determination. Extracts were heated at 60 °C for 5 min. Then, we added the rest of the buffer as instructed by the manufacturer. The mixture was vortexed and centrifuged at 14,000× *g* for 5 min before measuring at 450 nm. The obtained data were calculated based on the below equations provided by the manufacturer. In the NAD/NADH equation, ratio is the amount of total NAD (NAD+NADH) (pmole), whereas NADH is the amount of NADH in the sample (pmole) both from the standard curve. While for the CS equation, Sa is the amount of glutathione (nmole) generated in the sample between Tinitial and Tfinal from the standard curve. The Reaction Time is Tfinal − Tinitial (minutes); the sample volume (mL) and the data are reported as nmole/min/mL.

$$Ratio=\frac{NAD\;total\;–NADH}{NADH}$$

$$CS\;activity=\frac{Sa}{(Reaction\;Time)\times Sv}$$

### 2.3. Quantitative Real-Time PCR

Total RNA was extracted from frozen tissues using Trizol reagent (Thermo Fisher Scientific, Waltham, MA, USA) and an RNeasy Mini Kit (Qiagen, Germantown, MD, USA). Quantitative real-time PCR was performed with SYBR Green to measure *RRM2* expression levels and other genes mentioned in Table 1. RNA expression was normalized to *18S* rRNA expression using the 2^−ΔΔCt^ method to calculate fold change. The forward and reverse primers for PCR amplification are shown in Table 1.

### 2.4. Histology Analysis

Tissue samples were collected from the cranial end of each breast fillet and transferred to 10% paraformaldehyde (Sigma Aldrich, St. Louis, MO, USA) and fixed in paraffin wax [20]. Slides were prepared (perpendicular to the direction of the muscle fibers) using 8 µm sections and stained using Picrosirius red to measure muscle tissue damage. Staining procedures were completed by the University of Georgia, College of Veterinary Medicine, Histology Lab (Athens, GA, USA). Briefly, sections were stained with 0.1% Picrosirius red in saturated picric acid for a minimum of 1 h before dipping them in acidified water twice. All slides were dehydrated and mounted in Permount and then covered by cover slips. All images were obtained (20× magnification) using a light microscope (Zeiss AXIO, Imager.A2, Pleasanton, CA, USA) with the same exposure time, white balance, and saturation. All obtained images were analyzed using Fiji image software (ratio of scars or connective areas to the whole area).

### 2.5. Analysis

Student’s *t*-tests were used for comparison of the two groups using Prism (GraphPad software, San Diego, CA, USA, Vesrion 9.0). Results were considered significant at *p* ≤ 0.05. Mean values in the text are given as mean ± SE.

## 3. Results

### 3.1. Quantitative Real-Time PCR

**Figure 1:**
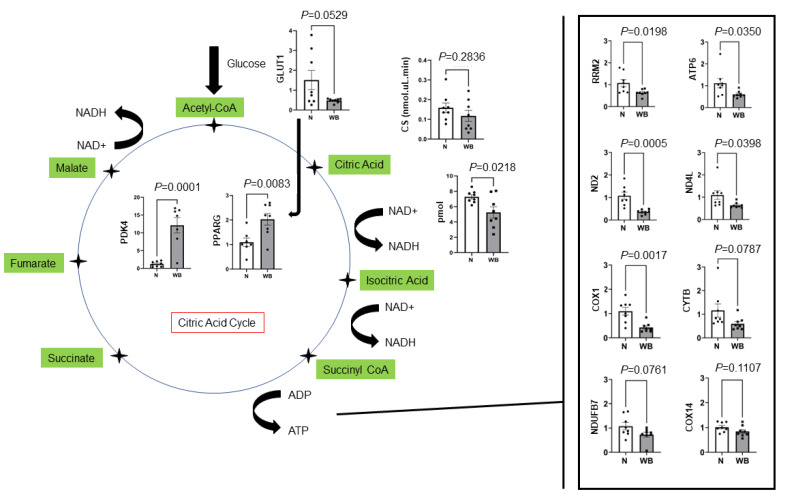
Changes in genes expression (fold change) in the citric acid cycle for woody (WB) and normal (N) breast muscles. *GLUT1*, citrate synthase activity (CS) and NAD/NADH reduced, whereas *PDK4* and *PPARG* both increased for WB. *ATP6*, *ND2*, *ND4L*, *COX1*, *CYTB* and *NDUFB7* reduced as potential factors responsible for ATP production and normal mitochondria function.

**Figure 2:**
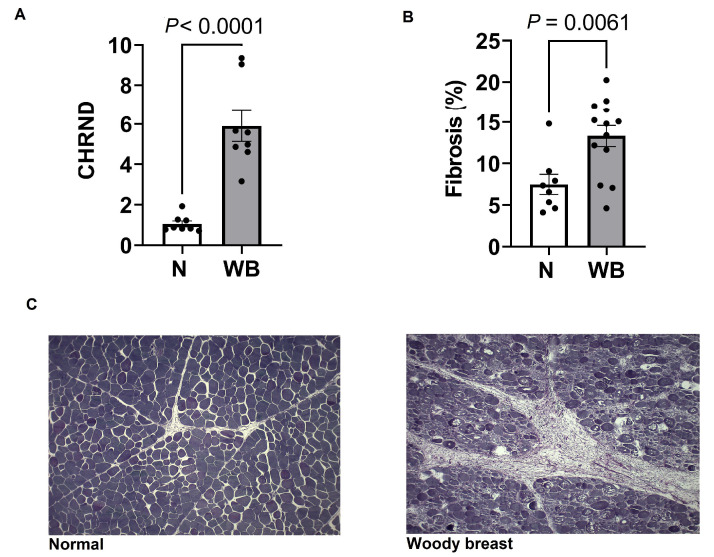
Changes in expression (fold change) of *CHRND* (**A**), and fibrosis (**B**) for severe woody (WB) and normal breast (N). Changes in connective tissues in normal (**C**, left) and WB (**C**, right).

Changes in the gene expression of multiple components of the citric acid cycle are presented in Figure 1. Results showed a decreased expression of *RRM2* (*p* = 0.01) in WB samples compared to N as well as several mtDNA genes required for normal mitochondria function, including *ATP6* (*p* = 0.03), *COX1* (*p* = 0.001), *CYTB* (*p* = 0.07), *ND2* (*p* = 0.0005) and *ND4L* (*p* = 0.03). Furthermore, *NDUFB7* (*p* = 0.07) and *COX14*, which are expressed in nuclear DNA and localized to mitochondria, tended to be lowered in WB compared to N. Additionally, WB muscle showed a decreased expression of *GLUT1* (*p* = 0.05) and increased expressions for *PDK4* (*p* = 0.0001) and *PPARG* (*p* = 0.008) as indicators of higher fatty acid oxidation. WB meat decreased the NAD/NADH ratio (*p* = 0.02, pmol), while it showed an increased expression of *CHRND* (*p* < 0.0001) (Figure 2).

### 3.2. Histology Analysis

WB increased fibrosis compared to N (*p* = 0.006) (Figure 2). WB tissue exhibited a greater ratio of connective tissues to normal cell muscle area and increased abnormalities to muscle cell structure and arrangement.

## 4. Discussion

**Figure 3:**
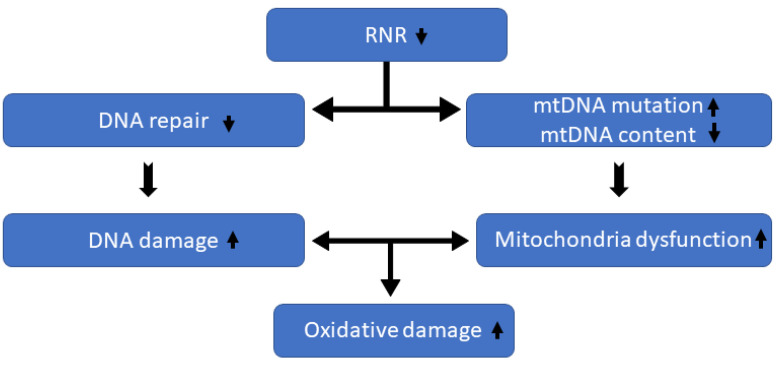
*RRM2* reduction impacts mitochondria DNA (mtDNA) health; consequently, it increases reactive oxygen species and oxidative damages.

The principal findings of this study indicated that *RRM2*, a subunit of RNR, may play roles by controlling mitochondria function, decreasing both ROS level and fibrosis. Figure 3 shows how disruptions in *RRM2* can increase oxidative stress.

A study [15] demonstrated that the disruption of *RRM2* impaired tissue health and mitochondria function by disrupting mtDNA synthesis, resulting in higher oxidative damage. We found that *RRM2* disruption could cause mitochondria dysfunction, resulting in impaired tissue health, indicating it could be related to woody breast. In addition, our results confirmed with a study [21] that both *CHRND* and fibrosis are good indicators of ROS level in tissues. Disorders such as severe infection or injuries may result in the up-regulation of nicotinic receptors by mitochondria, which contributes to an overabundance of receptors [22,23]. Fibrosis is related to the induction of apoptosis and changes in mitochondria membrane [24]. What followed is increased apoptosis as well as possible mitochondria dysfunction as evidenced by reduced several genes expression related to mtDNA and the reduced NAD/NADH ratio in this study. The NAD+/NADH ratio is associated with the cells’ metabolism; any alterations to the ratio are to be found in situations of metabolic diseases [25]. NAD+ is necessary for several enzymes’ activity related to metabolism. Consequently, a decrease in the ratio causes these enzymes to decrease in activity, resulting in an altered metabolic situation. Metabolomic alterations are related to the energy metabolism and mitochondria functionality in broiler chickens with severe woody breast [10]. In addition, the decrease in the genes’ expression related to mtDNA implies that DNA potentially is damaged, leading to lower ATP production. Therefore, when ATP production is negatively affected in the muscle, then the ability of muscle to regenerate is impaired. The abnormalities observed in this study for the WB group could be related to lower *RRM2* expression.

Furthermore, increased glucose oxidation/transport limits the oxidation of fatty acids by inhibiting their transport into the mitochondria [26]. The *GLUT* family proteins are the major players for glucose transport. Consequently, insufficient glucose availability, when transport is disrupted (*GLUT1* in this study), increases fatty acids in greater quantities to help the body produce energy (*PDK4* and *PPARG* in this study). Fatty acids represent an important source of energy in periods of catabolic stress; their oxidation produces acetyl-CoA, which supplies energy to other tissues when glycogen stores are depleted [27]. A study indicated that fatty acid oxidation links to the woody breast and a level of oxidative stress [6].

As mentioned earlier, RNR plays an important role in DNA replication and DNA repair processes [12,28] by controlling dNTPs during cell proliferation. Starvation for the DNA precursor could have a major impact on cell health [29], and missing a single DNA building block is sufficient to bring about cell death. Data have shown that *RRM2* acts as a molecular switch that affects RNR activity and DNA replication [12]. The mutation in *RRM2* leads to increased fibrosis in tissues [16], and altered *RRM2* has been associated with changes in mtDNA gene expression, ATP production, and ROS formation in tissues [15]. The top altered bio-functions in term of molecular and cellular functions in woody tissue included increased cell death and ROS generation [30]. Therefore, this study suggests that disruptions in RNR activity, as indicated by lowered *RRM2*, could potentially impact mitochondria function and increase woody breast development by impacting genes associated with mtDNA and CS.

## 5. Conclusions

The results of the current study suggest that *RRM2* appears to be associated with woody breast meat, and altering the *RRM2* expression might have adverse impacts on the meat quality. A reduction in *RRM2* appeared to have major impacts on tissue health and mitochondria function by increasing fibrosis while reducing the expression of genes related to mtDNA. As direct mechanistic connections between *RRM2* and woody breast have not been established, further studies are required to provide a better understanding of how *RRM2* is involved in woody breast development.

## Figures and Tables

**Table 1:** Forward and reverse primers for real-time PCR amplification.

Target ^1^	Forward	Reverse
***ATP6***	AATTCTCAAGCCCCTGCCTA	AGGAGGCCTAGGAGGTTAAT
***CHRND***	GTGGTCCTCAACTTCCATTTCC	AGGATCTCCAGGAACACCTCT
***COX14***	GGCTGATTTCGGCTACAAAGC	GCACAGGTACCCGCCGTA
***COX1***	TCCTTCTCCTACTAGCCTCA	AGGAGTAGTAGGATGGCAGT
***CYTB***	TGCCTCATGACCCAAATCCT	AGTGTGAGGAGGAGGATTACT
***GLUT1***	GCATGATCGGCTCCTTCTCTGT	AGCAGCGGCCAGAGAGAGTCGT
***ND2***	AGCATAACCAACGCCTGATC	GATGTTAGGAGGAGGAGTGT
***ND4L***	TCCCCTACACTTCAGCTTCT	TTCGCATGCTGAGAAGGCTA
***NDUFB7***	GACGCCTTCCCCAGCCTATG	CTCGCGCTCAAACTCCTTCAT
***PDK4***	TCTCCGCTCTCCATCAAGCA	TCTTGTCGCAGGAACGCAAA
***PPARG***	GTGCAATCAAAATGGAGCC	CTTACAACCTTCACATGCAT
***RRM2***	AGTGAGTGTGTATATGCTCCCC	CCAAAAGTCAAGGACGCTG
***18S*** ^2^	TCCCCTCCCGTTACTTGGAT	GCGCTCGTCGGCATGTA

^1^ *ATP6*: Mitochondria encoded ATP synthase membrane subunit 6; *CHRND*: Nicotinic acetylcholine receptor subunit delta; *COX14*: Cytochrome c oxidase assembly factor; *COX1*: Cytochrome c oxidase subunit 1 mitochondria gene; *CYTB*: Cytochrome b; *GLUT1*: Glucose transporter 1; *ND2*: NADH dehydrogenase 2; *ND4L*: Mitochondria encoded NADH:ubiquinone oxidoreductase core subunit 4L; *NDUFB7*: NADH:ubiquinone oxidoreductase subunit B7; *PDK4*: Pyruvate dehydrogenase kinase 4; *PPARG*: Peroxisome proliferator-activated receptor gamma; *RRM2*: Ribonucleotide reductase M2 subunit. ^2^ *18S*: housekeeping.

## Data Availability

No new data were created.
